# The inter-rater reliability and prognostic value of coma scales in Nepali children with acute encephalitis syndrome

**DOI:** 10.1080/20469047.2017.1398503

**Published:** 2017-11-16

**Authors:** Stephen Ray, Ajit Rayamajhi, Laura J. Bonnett, Tom Solomon, Rachel Kneen, Michael J. Griffiths

**Affiliations:** ^a^ Institute of Infection and Global Health, University of Liverpool, Liverpool, UK; ^b^ Littlewoods Neurosciences Unit, Alder Hey Children’s NHS Foundation Trust, Liverpool, UK; ^c^ National Institute for Health Research Health Protection Research Unit in Emerging and Zoonotic Infections, University of Liverpool, Liverpool, UK; ^d^ Department of Paediatrics, Kanti Children’s Hospital, Kathmandu, Nepal; ^e^ Department of Paediatrics, National Academy of Medical Sciences, Kathmandu, Nepal; ^f^ Department of Biostatistics, University of Liverpool, Liverpool, UK

**Keywords:** Acute encephalitis syndrome, coma scales, inter-rater reliability, prognostic value, AES, acute encephalitis syndrome, ACS, Adelaide coma scale, AVPU, alert, verbal, pain, unresponsive, BCS, Blantyre coma scale, ETAT, emergency triage assessment and treatment, LOS, Liverpool outcome score, NTBI, non-traumatic brain injury, PIM, paediatric risk of mortality, PRISM, paediatric risk of mortality score, RPS, resource-poor setting, TBI, traumatic brain injury

## Abstract

**Background:**

Acute encephalitis syndrome (AES) is a common cause of coma in Nepali children. The Glasgow coma scale (GCS) is used to assess the level of coma in these patients and predict outcome. Alternative coma scales may have better inter-rater reliability and prognostic value in encephalitis in Nepali children, but this has not been studied. The Adelaide coma scale (ACS), Blantyre coma scale (BCS) and the Alert, Verbal, Pain, Unresponsive scale (AVPU) are alternatives to the GCS which can be used.

**Methods:**

Children aged 1–14 years who presented to Kanti Children’s Hospital, Kathmandu with AES between September 2010 and November 2011 were recruited. All four coma scales (GCS, ACS, BCS and AVPU) were applied on admission, 48 h later and on discharge. Inter-rater reliability (unweighted kappa) was measured for each. Correlation and agreement between total coma score and outcome (Liverpool outcome score) was measured by Spearman’s rank and Bland–Altman plot. The prognostic value of coma scales alone and in combination with physiological variables was investigated in a subgroup (*n* = 22). A multivariable logistic regression model was fitted by backward stepwise.

**Results:**

Fifty children were recruited. Inter-rater reliability using the variables scales was fair to moderate. However, the scales poorly predicted clinical outcome. Combining the scales with physiological parameters such as systolic blood pressure improved outcome prediction.

**Conclusion:**

This is the first study to compare four coma scales in Nepali children with AES. The scales exhibited fair to moderate inter-rater reliability. However, the study is inadequately powered to answer the question on the relationship between coma scales and outcome. Further larger studies are required.

## Introduction

Acute encephalitis syndrome (AES) is defined as a person of any age at any time of year with acute onset of fever and a change in mental status (including symptoms such as confusion, disorientation, coma or inability to talk) AND/OR new onset of seizures (excluding simple febrile seizures) [1]. The most commonly identified cause of AES in Nepali children is the Japanese encephalitis (JE) virus which accounts for around a quarter to one-third of cases [2,3]. However, the syndrome can be associated with a range of pathogens, including acute bacterial or parasitic infection [3]. In most cases, no pathogen is identified and management is supportive [2]. The syndrome is a common cause of non-traumatic brain injury (NTBI) in children in resource-poor Asian countries with high morbidity and mortality [4]. Historically, the Glasgow coma scale (GCS) was designed to assess the level of impaired consciousness in traumatic brain injury (TBI) [5]. It has since been adopted for use in NTBI. Despite conflicting evidence, GCS is used to assess disease severity and clinical outcome in encephalitis [6–8]. Alternative coma scales may have better inter-rater reliability and prognostic value in encephalitis, but this has not been studied [5]. The Adelaide coma scale (ACS), the Blantyre coma scale (BCS) and the alert, verbal, pain, unresponsive (AVPU) scale are alternative coma tools which can be used in children.

Complex clinical scoring systems such as the paediatric risk of mortality score (PRISM) or the paediatric risk of mortality score (PIM) are used to calculate mortality risk in paediatric intensive care in resource-rich settings. These are reported to predict clinical outcome more accurately than coma or composite (coma and physiological) clinical decision tools [5]. However, these tests are labour intensive and often not appropriate in resource-poor settings (RPS) where there is limited intensive care support and 50% of childhood deaths occur within the first 24 h in hospital [9]. A simplified clinical scoring system is required for use in real practice in the RPS setting. Extra investment to triage unwell children with reduced consciousness is frequently not available [10]. In response to these challenges, an emergency triage assessment and treatment (ETAT) system for children has been developed in Africa. This is based on rapid assessment of heart rate, respiratory rate, hydration status and consciousness level and enables appropriate emergency care.

The inter-rater reliability and prognostic value of four coma scales (GCS, ACS, BCS and AVPU) were assessed when applied on their own and in combination with physiological parameters in children with AES in Kathmandu [3].

## Methods

Children aged 1–14 years who presented to Kanti Children’s Hospital, Kathmandu between September 2010 and November 2011 and who fulfilled the clinical criteria for AES based on the World Health Organization’s definition [11] were prospectively recruited. Assessment was as follows: each coma scale (GCS, ACS, BCS and AVPU) was applied on admission, 48 h later and on discharge (Table 1). The GCS rather than the paediatric GCS was applied because the former is used routinely in the hospital.

**Table 1.** Coma scales used in the study.

Blantyre coma scale		Glasgow coma scale	Adelaide paediatric coma scale			AVPU	
*Eye response*							
Directed eye movement	1	Spontaneous	4	Spontaneous	4	Alert	4
Not directed	0	To speech	3	To speech	3	Voice	3
		To pain	2	To pain	2	Pain	2
		None	1	None	1	Unresponsive	1
*Best verbal response*							
Appropriate cry	2	Oriented	5	Oriented	5		
Inappropriate cry/moan	1	Confused	4	Words	4		
No cry	0	Inappropriate words	3	Vocal sounds	3		
		Incomprehensible sounds	2	Cries	2		
		None	1	None	1		
*Best motor response*							
Localises pain	2	Obeys	6	Obeys commands	5		
Withdraws from pain	1	Localises	5	Localises pain	4		
No response	0	Withdraws	4	Flexion to pain	3		
		Abnormal flexion	3	Extension to pain	2		
		Extensor response	2	None	1		
		None	1				
Total 0–5		Total 3–15		Total 3–14		Total 1–4	

Three pairs of general paediatric clinicians independently documented scores on each coma scale. Two pairs were treating clinicians involved in the care of the patients, and the other pair was research clinicians who were not involved in the care of the patients. The second observer in each pair applied the scale immediately after the first to minimise temporal variation in consciousness level. At discharge, to complete the Liverpool outcome score (LOS), a validated outcome score for assessing functional impairment in children with AES [12], the child was examined clinically and the family was interviewed.

### Statistical analysis

Inter-rater reliability was measured by unweighted kappa (*κ*). The kappa scores were interpreted following published guidelines: *κ* = 0, response probably owing to chance; 0.01–0.2, slight agreement; 0.21–0.40, fair agreement; 0.41–0.60, moderate agreement; 0.61–0.80, substantial agreement; >0.81, almost perfect agreement [13]. Data were analysed using PRISM version 6.

Agreement between total LOS and each summated coma score on admission was measured by generating a Bland–Altman plot and computing the limits of agreement [95% confidence interval (95% CI)]. Correlation between LOS and each coma score was assessed using Spearman’s rank correlation coefficient.

The patients’ clinical records (*n* = 50) were assessed to investigate if there were indicators of poor outcome. The ability of the admission GCS to predict outcome in a patient subgroup (*n* = 22) with available data for physiological parameters (respiratory rate, heart rate, blood pressure) was assessed via *t*-tests or Mann–Whitney U tests in the case of non-normally distributed data.

To identify which features were independently associated with poor outcome, these physiological variables and coma scores were entered into a multivariable logistic regression model with variable selection via backward selection. Data were analysed using SPSS.

Patients were additionally split into two groups based on a GCS score (≤8 or >8) and the sensitivity, specificity and accuracy of poor outcome prediction were assessed. This threshold was chosen because ≤8 is reported to indicate severely impaired consciousness requiring intubation [5,14]. The ability of combining GCS with systolic blood pressure to predict outcome was assessed in terms of sensitivity, specificity and accuracy; a GCS ≤8 and <91 systolic blood pressure (the 5th–50th centile for this physiological parameter in our study cohort) [14].

### Ethics

Ethics approval was granted by the Institutional Review Committee of Kanti Children’s Hospital. Written informed consent was obtained from the parents or guardians of all study participants.

## Results

**Figure 1.**
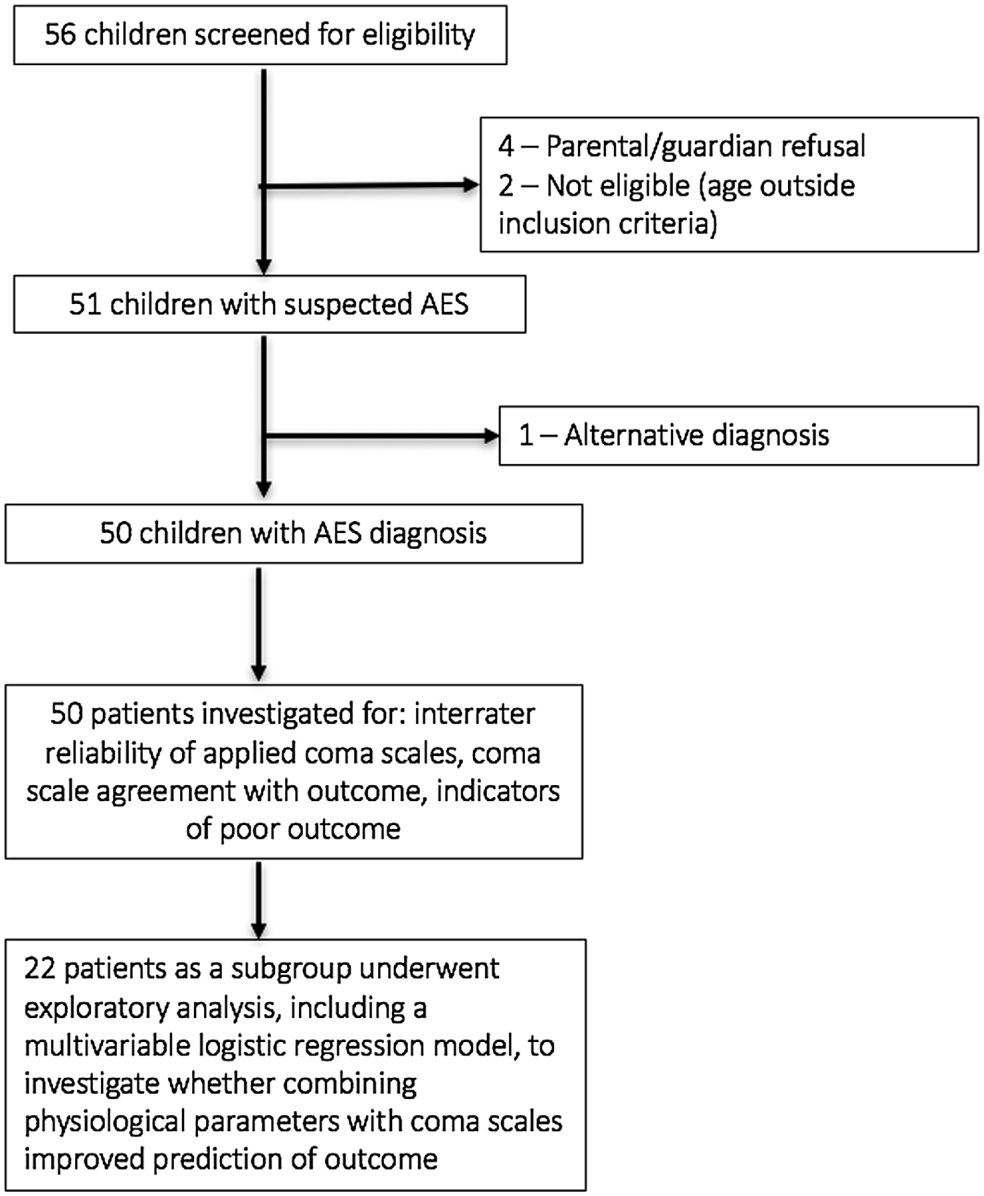
Study flowchart.

Of the 56 children screened, six were excluded, leaving 50 children with AES (aetiology unknown) in the study (Figure 1). Males were 62% of participants, median age was 6 years (range 1–15) and seizures were present in five (10%) cases. The inter-rater reliability of each coma scale was compared using the mean score (data followed Gaussian distribution) over three time-points (admission, 48 h later and discharge) from each observer. Three scores showed moderate agreement: ACS (0.52), GCS (0.53) and AVPU (0.58). The BCS showed fair agreement (0.37).

**Figure 2.**
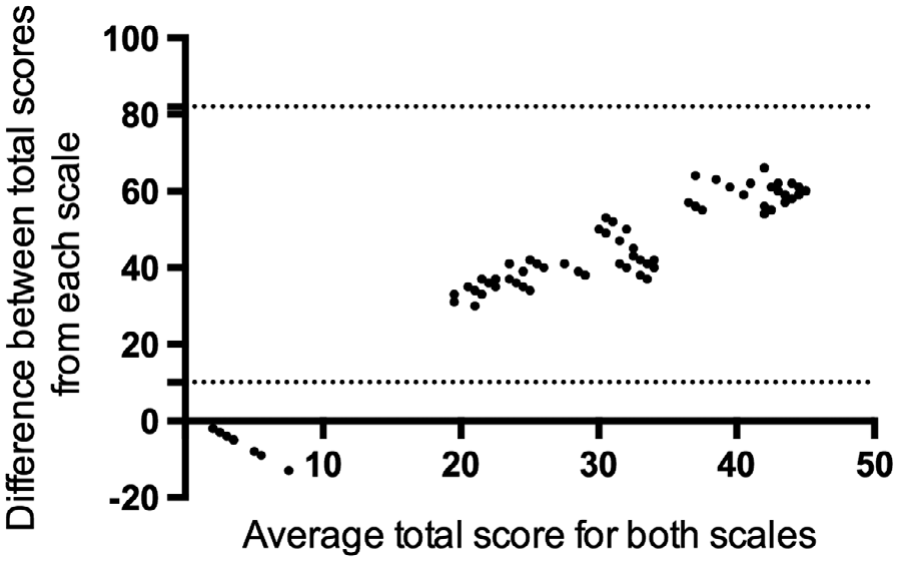
Bland–Altman plot measuring agreement between total Glasgow coma score and outcome (Liverpool outcome score). The plot displays mean (*X* axis) and difference (*Y* axis) in the total LOS (scored on discharge) and total GCS scores (scored on admission) in child AES patients (*n* = 50). Dotted lines demarcate the limits of agreement (±2 standard deviations from the mean difference). Forty-three children had scores for both the LOS and GCS within the limits of agreement. Seven children plotted below the lower limit of agreement.

Admission GCS and discharge LOS exhibited a reasonable level of agreement; 43 children (86%) displayed scores for both GCS and LOS within the 95% CI limit of agreement. However, seven children (14%) exhibited poor agreement, plotting outside the 95% CI (Figure 2). These latter patients all exhibited a high admission GCS but low discharge LOS. All seven patients died within 48 h of admission.

Next, coma scores at each of the three time-points were correlated against total LOS for each scale: admission (GCS 0.70, ACS 0.68, BCS 0.69, AVPU 0.71), 48 h (GCS 0.74, ACS 0.74, BCS 0.75, AVPU 0.75) and discharge (GCS 0.78, ACS 0.81, BCS 0.77, AVPU 0.77).

Correlation between discharge LOS and coma scores were weaker at admission compared to later time-points. The patients identified as outliers through the Bland–Altman plot again influenced the correlations. Removing the same seven patients in a sensitivity analysis, admission coma scores exhibited stronger correlations with total LOS (GCS 0.77, ACS 0.76, BCS 0.76, AVPU 0.80), more comparable with the correlations for the later time-points.

The children who died (*n* = 7) were more likely to be transferred to paediatric intensive care (deaths 83.3% *vs* survivors 14.3%, *p* = 0.005) or to receive a higher number of drugs during admission despite a shorter inpatient time (median number of drugs 4.7 *vs* 3.0, deaths *vs* survivors *p* = 0.09).

Examining a sub-group of AES patients (*n* = 22) with available data for respiratory rate, heart rate, blood pressure and age on admission, physiological parameters were compared between those who died (*n* = 4) and survivors (*n* = 18). Patients who died had lower systolic blood pressure and respiratory rate (*p* = 0.04 and *p* = 0.06, respectively, Table 2).

**Table 2.** Physiological parameters, GCS score and Liverpool outcome score in paediatric AES patients (*n* = 22).

	Alive, *n* = 18 Median (range) [No. of patients]	Dead, *n* = 4 Median (range) [No. of patients]	*p*–value*
Days since onset	5 (3–13) [11]	4 (3–14)	0.72
Admission GCS	12 (4–15)	4 (3–10)	**0.02**
LOS	69 (46–75)	1 (1)	**0.00**
Age (years)	7.0 (1–13)	11.5 (9–14)	0.06
Systolic blood pressure	110 (80–125) [15]	86 (80–90) [3]	**0.04**
Respiratory rate	35 (20–68)	17 (12–40)	0.06
Heart rate	108 (80–120)	111 (65–138)	0.67
Temperature	31.2 (29.2–37.2)	38.9 (36.7–39.4) [3]	0.05

Notes: *p-values in bold are statistically significant. Significance of difference between groups measured by *t*-test or Mann–Whitney U-test. GCS, GCS (score 3–15); LOS, Liverpool Outcome Score (1 [died] – 75 [no impairment]).

The multivariable model included the GCS on admission and systolic blood pressure (Table 3). A complete case analysis as a sensitivity analysis showed consistent results.

**Table 3.** Multivariable logistic regression model with variable selection via backward selection.

Variable	Univariable *p*-value*	Univariable OR (95% CI)	Multivariable *p*-value*	Multivariable OR (95% CI)
Admission GCS	**0.01**	0.67 (0.38–0.93)	0.06	0.68 (0.34–1.02)
Heart rate	0.64	0.99 (0.93–1.05)	N/A	N/A
Respiratory rate	**0.03**	0.87 (0.71–0.99)	N/A	N/A
Blood pressure	**0.03**	0.82 (0.58–0.98)	**0.02**	0.78 (0.46–0.98)

Notes: *p-values in bold are statistically significant. OR, odds ratio; CI, confidence interval; N/A, dropped from multivariable model during backward selection.

Low GCS (≤8) on admission correctly predicted three out of four deaths (75% sensitivity), 14 of 18 survivors (78% specificity), and correctly classified 17 of 22 patients as going to die or survive (77% accuracy).

Combining the GCS with systolic blood pressure (independently associated with poor outcome by the multivariable model) correctly predicted two out of three deaths (67% sensitivity), 14 out of 15 survivors (93% specificity), and correctly classified 16 of 18 patients as going to die or survive (89% accuracy).

## Discussion

Coma scales (GCS, ACS, AVPU) exhibited moderate (*κ* 0.41–0.60) agreement between observers when applied to Nepali children with AES. These kappa scores reflect previous reports for inter-rater agreement when applied to children with cerebral malaria [15]. On admission, the coma scales were poor predictors of clinical outcome. Although studies in TBI demonstrate that coma scores used in isolation can accurately predict outcome [16], previous reports in NTBI are in keeping with our findings that coma scales are poor predictors of clinical outcome [3–5]. The ETAT tool which measures heart rate, respiratory rate, hydration status and consciousness level on admission facilitates appropriate emergency care in the resource-poor setting; in Malawi, it has halved inpatient mortality [9]. In this study, combining GCS with physiological parameters such as systolic blood pressure improved outcome prediction when analysed by a logistic regression model.

The authors have previously reported that Nepali children suffering AES who exhibit a low respiratory rate tend to have a poor outcome. In contrast, those with a higher respiratory rate (median 30 bpm) tend to have a good outcome [3]. A raised respiratory rate may indicate a compensatory response to fever and/or dehydration. In contrast, a relatively low respiratory rate and blood pressure when a child has a fever may reflect a lack of appropriate physiological compensation.

In the current study, the Nepali children who later died exhibited respiratory rates and heart rates within the normal range for their age [14]. Like any clinical tool, ETAT may need to be adapted for use in other populations such as in Nepal so that children with AES and fever but a relatively low respiratory rate, blood pressure and heart rate trigger concern. Additionally, GCS is the standard coma scale used in Nepal (rather than AVPU used in ETAT), so this may also need to be amended into an adapted version of ETAT for AES.

Clinical decision tools such as ETAT could enable systematic assessment of illness severity among children with AES. Since the tools do not require expensive equipment, they are also financially appealing in countries where AES is prevalent.

The study has a number of limitations. The incidence of AES was lower than expected during study recruitment (based on previous hospital data). This contributed to the small study sample size. In addition, a lack of detailed medical documentation, e.g. blood pressure either prospectively collected on the ward during admission or when examined retrospectively in medical records, prevented assessment of coma scales combined with physiological parameters in all AES patients.

A larger study with ETAT or similar composite clinical scores applied prospectively to children with AES is recommended.

This is the first study to compare four coma scales in Nepali children with AES. The scales exhibited fair-to-moderate inter-rater reliability. Combining coma scores, e.g. GCS with other physiological parameters such as systolic blood pressure, may improve outcome prediction. However, the modest sample size prevents these results being adequately powered to answer the question on the relationship between coma scales and outcome. One may speculate that a clinical decision tool measuring coma severity in combination with other physiological markers may improve identification of children with AES at risk of death. This could potentially help guide provision of earlier supportive treatments among AES previously associated with improved outcome [3]. However, further larger studies are required to adequately investigate this.

## Disclosure statement

No potential conflict of interest was reported by the authors.

## Funding

Stephen Ray is funded by a Wellcome Trust Training Fellowship [grant number 203919/Z/16/Z]. Laura Bonnett is funded by a post-doctoral fellowship from the National Institute for Health Research [grant number PDF-2015-08-044].

## Notes on contributors

***Stephen Ray*** is a paediatric registrar and holds a Wellcome Trust Tropical Clinical PhD Training Fellowship. His research interests include the aetiology of paediatric febrile coma in sub-Saharan Africa and using host response markers to differentiate between different paediatric central nervous system infections in resource poor settings.

***Ajit Rayamajhi*** is an associate professor and consultant paediatrician in Kathmandu, Nepal. His research interests include acute encephalitis syndrome, especially improving the prevention, diagnosis and treatment of Japanese Encephalitis.

***Laura J. Bonnett*** is a statistician and health research postdoctoral research fellow in National Institute. Her Research interests include statistical modelling, in particular estimating time to next event for patients with recurrent conditions such as epilepsy and asthma.

***Tom Solomon*** is a professor, head of the Brain Infections Group and honorary consultant neurologist. His research interests include central nervous system infections, especially flaviviruses and emerging infections.

***Rachel Kneen*** is a consultant paediatric neurologist and honorary senior clinical lecturer. Her research interests include paediatric central nervous system infections, especially encephalitis, plus the genetics underlying paediatric epilepsy.

***Michael J. Griffiths*** is a senior clinical lecturer and honorary consultant paediatric neurologist. His research interests include paediatric brain infections in the UK and globally, especially the host response to severe infection, with a focus on identifying diagnostic and prognostic markers.
